# Pharmacological Activation Of Aldehyde Dehydrogenase 2 Protects Against Heatstroke-Induced Acute Lung Injury by Modulating Oxidative Stress and Endothelial Dysfunction

**DOI:** 10.3389/fimmu.2021.740562

**Published:** 2021-10-26

**Authors:** Hsiao-Ya Tsai, Yu-Juei Hsu, Cheng-Yo Lu, Min-Chien Tsai, Wan-Chu Hung, Po-Chuan Chen, Jen-Chun Wang, Lung-An Hsu, Yung-Hsin Yeh, Pauling Chu, Shih-Hung Tsai

**Affiliations:** ^1^ Department of Emergency Medicine, Tri-Service General Hospital, National Defense Medical Center, Taipei, Taiwan; ^2^ Division of Nephrology, Department of Internal Medicine, Tri-Service General Hospital, National Defense Medical Center, Taipei, Taiwan; ^3^ Center for the Prevention and Treatment of Heat Stroke, Tri-Service General Hospital, National Defense Medical Center, Taipei, Taiwan; ^4^ Department of Physiology and Biophysics, Graduate Institute of Physiology, National Defense Medical Center, Taipei, Taiwan; ^5^ Cardiovascular Department, Chang-Gung Memorial Hospital and School of Medicine, Chang-Gung University, Taoyuan, Taiwan

**Keywords:** heatstroke, heat stress, aldehyde dehydrogenase 2 (ALDH2), Alda-1, acute lung injury (ALI), reactive oxygen species (ROS)

## Abstract

Heatstroke (HS) can cause acute lung injury (ALI). Heat stress induces inflammation and apoptosis *via* reactive oxygen species (ROS) and endogenous reactive aldehydes. Endothelial dysfunction also plays a crucial role in HS-induced ALI. Aldehyde dehydrogenase 2 (ALDH2) is a mitochondrial enzyme that detoxifies aldehydes such as 4-hydroxy-2-nonenal (4-HNE) protein adducts. A single point mutation in ALDH2 at E487K (ALDH2*2) intrinsically lowers the activity of ALDH2. Alda-1, an ALDH2 activator, attenuates the formation of 4-HNE protein adducts and ROS in several disease models. We hypothesized that ALDH2 can protect against heat stress-induced vascular inflammation and the accumulation of ROS and toxic aldehydes. Homozygous ALDH2*2 knock-in (KI) mice on a C57BL/6J background and C57BL/6J mice were used for the animal experiments. Human umbilical vein endothelial cells (HUVECs) were used for the *in vitro* experiment. The mice were directly subjected to whole-body heating (WBH, 42°C) for 1 h at 80% relative humidity. Alda-1 (16 mg/kg) was administered intraperitoneally prior to WBH. The severity of ALI was assessed by analyzing the protein levels and cell counts in the bronchoalveolar lavage fluid, the wet/dry ratio and histology. ALDH2*2 KI mice were susceptible to HS-induced ALI *in vivo*. Silencing ALDH2 induced 4-HNE and ROS accumulation in HUVECs subjected to heat stress. Alda-1 attenuated the heat stress-induced activation of inflammatory pathways, senescence and apoptosis in HUVECs. The lung homogenates of mice pretreated with Alda-1 exhibited significantly elevated ALDH2 activity and decreased ROS accumulation after WBH. Alda-1 significantly decreased the WBH-induced accumulation of 4-HNE and p65 and p38 activation. Here, we demonstrated the crucial roles of ALDH2 in protecting against heat stress-induced ROS production and vascular inflammation and preserving the viability of ECs. The activation of ALDH2 by Alda-1 attenuates WBH-induced ALI *in vivo*.

## Highlights

• Mice carrying the human ALDH2*2 variant were susceptible to whole-body heating-induced acute lung injury.
• Silencing ALDH2 induced 4-HNE and ROS accumulation in endothelial cells subjected to heat stress.
• Alda-1 attenuated the heat stress-induced activation of inflammatory pathways, senescence and apoptosis *in vitro*.
• Activation of ALDH2 by Alda-1 attenuated whole-body heating-induced acute lung injury *in vivo*.

## Introduction

Heat-related illness (HRI) affects a large number of people and is an increasing cause of health issues, as climate change results in elevated global temperatures (1, 2). The heatstroke (HS) -related inflammatory response is akin to the systemic inflammatory response syndrome and lead to a rapid deterioration in clinical status, resulting in disseminated intravascular coagulation, acute lung injury (ALI), multiorgan failure syndrome (MODS) and death (3, 4). Heat stress induces several inflammatory and apoptotic pathways and increases the production of reactive oxygen species (ROS) and endogenous reactive aldehydes (5–9). As an inducible transcription factor, nuclear factor-kappa B (NF-κB) can be activated by ROS, cytokines, and endotoxin and is associated with the pathophysiological changes associated with heat stress and strenuous exercise (10–12). Endothelial cells (ECs) play an essential role in maintaining the stability of microvascular permeability. Heat stress induces cellular senescence, apoptosis and pyroptosis in a variety of cell types, and endothelial activation/dysfunction with hyperpermeability play crucial roles in HS-induced ALI (13–17). The accumulation of toxic aldehydes and oxidative stress and upregulation of the NF-κB signaling pathway have been found in the hippocampus and lung tissues of rats subjected to HS (18, 19). Scavenging ROS significantly inhibited HS-induced necroptosis, suggesting that preventing necroptosis could alleviate HS-induced small intestinal tissue injury and cell death (20).

Mitochondrial aldehyde dehydrogenase 2 (ALDH2) is an enzyme that detoxifies aldehydes by converting toxic exogenous and endogenous aldehydes such as 4-hydroxy-2-nonenal (4-HNE) protein adducts and lipoperoxides such as malondialdehyde (MDA) to form nontoxic carboxylic acids. Although ALDH2 was initially known for its crucial role in ethanol metabolism in the liver, it has since been implicated in a variety of diseases, such as cardiovascular diseases (CVDs), diabetes, neurologic dysfunctions and ischemia reperfusion injury (IRI), in several organs (21–25). Endogenous aldehydic products, such as 4-HNE and MDA, can be formed by lipid peroxidation of mitochondrial and plasma membranes under oxidative stress conditions (26, 27). ALDH2 deficiency is known to increase oxidative stress due to an imbalance in antioxidant defense and ROS generation (8, 28). A single point mutation in ALDH2 at E487K, which is known as ALDH2*2, intrinsically lowers ALDH2 activity in approximately 40% of East Asian individuals. The ALDH2 activator Alda-1 binds to ALDH2 and restores ALDH2 activity by acting as a structural chaperone (29). Previous studies indicated that Alda-1 attenuated the formation of 4-HNE protein adducts and inactivated the NF-κB pathway in several disease models (30–34). Pretreatment with Alda-1 had been shown to have beneficial effects on hyperoxia and acrolein induced ALI through preserving the endothelial barrier and mitochondrial dysfunction (35–37).

While ALDH2 protects against oxidative damage through the oxidation of toxic aldehydes, few studies have investigated the role of ALDH2 in the pathogenesis of HS. We hypothesized that ALDH2 can protect against heat stress-induced vascular inflammation and ROS and the accumulation of toxic aldehydes. We further tested whether the ALDH2 activator Alda-1 could be an adjunctive therapy for HS-induced ALI.

## Materials and Methods

### Cell Culture and Reagents

Human umbilical vein endothelial cells (HUVECs) were obtained from Cell Applications (San Diego, CA, USA) and Taiwan Medical Cell and Bioresource Collection and Research Center (BCRC, Taiwan). Cells at passages 3-5 were used for the experiments with ALDH2-silencing RNA (siALDH2, Santa Cruz, sc-60147) and Alda-1 (Adooq Bioscience, A15805, 20 mM in DMSO as stock solution, 1:1,000 dilution (20μM) for the *in vitro* experiments). According to previous published literatures and our own experiences (24–26, 38), we believe that DMSO in such concentration would not exert obvious toxic or protective effects on endothelial cells. HUVECs were cultured in M200 medium supplemented with endothelial growth factor (Gibco, Medium 200 and LSGS) and maintained in a humidified atmosphere at 37°C and 5% CO_2_. The control cells were maintained in an incubator at 37°C. For heat stress induction, cells were subjected to 42°C for 2 h and then at 37°C overnight, as previously described (11, 15).

### Immunoblotting

Protein lysates from the cells and lung tissues were subjected to SDS-PAGE followed by electroblotting onto PVDF membranes. The membranes were probed with monoclonal antibodies against ALDH2 (Abcam, ab108306), cytochrome c (Santa Cruz, sc-7159), caspase 3 (CST, #9662), 4-HNE (Abcam, ab46545), p-p38 (CST, #9215), p38 (CST, #9212), p-p65 (CST, #3033), p65 (CST, #3034), NOX1 (GeneTex, GTX103888), NOX4 (GeneTex, GTX121929) and GAPDH (Santa Cruz, sc-32233). Bands were visualized by chemiluminescence detection reagents, and densitometric analysis was conducted with imaging processing software (Multi Gauge, Fujifilm). These data are expressed as the fold changes relative to the controls.

### Determination of ALDH2 Activity

ALDH2 activity was measured using an ALDH2 activity assay kit according to the manufacturer’s protocol (Abcam, ab115348, Cambridge, UK). In brief, the activity was estimated by measuring the conversion of oxidized nicotinamide adenine dinucleotide (NAD^+^) to reduced nicotinamide adenine dinucleotide (NADH) at an absorbance of 450 nm every 5 minutes for 2 hours period in the lung tissues and every 30 minutes for a 6 hours period (39).

### Measurement of ROS

ROS measurement was performed according to the manufacturer’s recommendations (OxiSelecte *in vitro* ROS/reactive nitrogen species (RNS) assay kit, Green Fluorescence; Catalog #STA-347, Cell Biolabs, Inc., San Diego, CA, USA). This *in vitro* assay measured total ROS/RNS free radical activity. Unknown ROS or RNS samples or standards were added to the wells with a catalyst that helps accelerate the oxidative reaction. Samples were measured fluorometrically against hydrogen peroxide. The free radical content in the samples was determined by comparison with a hydrogen peroxide standard curve. In brief, the cell lysates were stained with 2’,7’-dichlorofluorescein diacetate (DCFH-DA), which is oxidized by ROS to form fluorescent 2’,7’-dichlorofluorescein and were measured at an excitation wavelength of 488 nm and an emission wavelength of 535 nm. In addition, the dihydroethidium (DHE) method was also used to detect superoxide production at an excitation wavelength of 518 nm and an emission wavelength of 606 nm. The samples were loaded onto black 96-well plates and incubated for 30 min at 37°C, and the relative fluorescence units (RFUs) were determined by a fluorescence microplate reader (BMG Labtech, Ortenberg, Germany).

### ALDH2*2 Gene-Targeted Knock-In Mice

Knock-in mice on a C57BL/6J background with an inactivating point mutation in ALDH2 (ALDH2*2) were generated by homologous recombination, as previously described (34). There were no significant phenotypic changes in ALDH2*2 KI mice. Homozygous ALDH2*2 KI mice were used for the experiments.

### Murine Model of Whole-Body Hyperthermia

C57BL/6J mice and homozygous ALDH2*2 KI mice with a C57BL/6J background were used for the animal experiments. The mice were directly exposed to whole-body heating (WBH) at 42°C for 1 h at 80% relative humidity using a temperature-controlled environmental chamber from room temperature and then returned to room temperature for 6-hour recovery period. Rectal temperature was measured using a copper-constantan thermocouple probe inserted into the rectum and connected to a thermometer. After the 1-h heating period, the mice were returned to their home cages and given food and water ad libitum (40). These mice were treated with either vehicle control (20% DMSO and 20% PEG 400 in 100μl PBS) or Alda-1 [16 mg/kg in 100 μl in PBS with 20% DMSO and 20% PEG 400 (Sigma, 06855)] intraperitoneally 30 minutes prior to WBH as previously described with some modification (34, 41). Previous literatures had showed that there were no obvious toxic or protective effects of this DMSO preparation on animal experiments (34, 42). Mice were considered adequately anesthetized when no attempt to withdraw the limb after pressure was observed. At the end of the study, the mice were euthanized by exsanguination under anesthesia. Bronchioalveolar lavage fluid (BALF) was collected at the end of the experiment by slowly irrigating the right lung with two separate 0.7-ml aliquots of PBS, of which 1.2 ml could be retrieved consistently. To avoid overdistention, the pressure should be kept less than 20 mmH_2_O. All experimental protocols and procedures were approved by the Institutional Animal Care Committee of the National Defense Medical Center (Taipei, Taiwan) (43).

### Assessment of ALI

Lungs were collected after the mice were sacrificed following WBH. The wet weights of the organs were measured, and their dry weights were determined after the tissues were fully dried in an oven at 105°C. The water content was calculated as a percentage according to the following formula: 100×(wet weight-dry weight)/wet weight. One BALF aliquot was used immediately to measure the total cell counts. Erythrocytes were lysed using erythrocyte lysis buffer (Sigma, 1814389001), and the BALF was centrifuged at 400 g for 5 min and the supernatant was discarded. The pelleted cells were resuspended in 1.0 ml of PBS for the total leukocyte count by using a hemocytometer as previously described (44). The protein concentration in the supernatant was determined using bicinchoninic acid (BCA) method (Pierce, Rockford, IL, USA).

### Histology and Immunohistochemistry

Lung injury was evaluated by histological analysis as described previously. In brief, lung tissue was fixed in 10% formalin solution for 24 h and stained with hematoxylin and eosin (H&E). Lung injury was scored based on (1) the infiltration or accumulation of neutrophils in the airspace or vessel wall and on (2) the thickness of the alveolar wall. These two observations were scored from 0 (normal) to 5 (most severe injury or greatest thickness, respectively) (45).

### Cell Viability

Cell proliferation was analyzed by the MTT assay (Sigma, #11465007001) in accordance with the manufacturer’s protocols. Briefly, 5,000 cells/well were grown in 96-well plates, exposed to heat stress at 42°C for 2 h and recovered at 37°C overnight. Then, the cells were incubated with MTT medium in a 5% CO_2_ incubator at 37°C. After 4 h, the absorbance was measured at 570 nm using a Clariostar microplate reader (BMG Labtech, Ortenberg, Germany).

### Terminal Deoxynucleotidyl Transferase-Mediated dUTP Nick End-Labeling Assay

Cell apoptosis was evaluated by a TUNEL assay using a fluorescein direct *in situ* apoptosis detection kit (Millipore, S7110) according to the manufacturer’s recommended protocol. Apoptosis was determined as the percentage of positive cells per 1,000 DAPI-stained nuclei, and the cells were visualized under a fluorescence microscope (Nikon Eclipse 50i) at a magnification of 100×.

### Senescence Assay

Cellular aging was assessed with a senescence cell staining kit according to the manufacturer’s instructions (Sigma, CS0030). Cultured HUVECs were fixed and then incubated with fresh X-gal staining solution (1 mg/ml, 5 mmol/l potassium ferrocyanide, 5 mmol/l potassium ferricyanide, and 2 mmol/l MgCl_2_; pH 6). After the cells were stained, the numbers of blue-stained and total cells were determined, and the percentage of β-galactosidase-positive cells was calculated.

### Assessment of Mitochondrial Injury

The mitochondrial membrane potential (ΔΨm) has been used as a parameter of mitochondrial function (46). To assess mitochondrial function in HUVECs after HS, JC-1 (5,5’,6,6’-tetrachloro-1,1’,3,3’- tetraethylbenzimidazolcarbocyanine iodide) staining (BD Biosciences, 551302) was performed and assessed by fluorescence microscopy (Nikon Eclipse 50i) at a magnification of 200×. The images represent the merging of red and green channels. In addition, the fluorescence intensity of the cells was measured by a fluorescence microplate reader (BMG Labtech, Ortenberg, Germany). The data are expressed as the ratio of red fluorescence to green fluorescence intensity.

### Serum Levels of Organ Injuries

The serum levels of creatine kinase (CK), aspartate transferase (AST), and blood urea nitrogen (BUN) were measured by a FUJI Dri-chem slide on a FUJIDRI-CHEM 4000i instrument.

### Statistical Analysis

All experiments were performed independently at least 3 times, and all continuous variables are presented as the mean ± standard deviation (SD). The F test for equal variance was performed before the differences among groups were analyzed. Comparisons between two groups were analyzed using Student’s t test. For multiple groups, the data were analyzed using one-way ANOVA. For *post hoc* analysis, the Tukey test was used to correct for multiple comparisons, and the Fisher Least Significant Difference test was used for planned comparisons. Target protein expression measured by immunoblotting was analyzed by densitometry and is expressed as percent changes relative to an internal control or as the phosphorylated protein level relative to the total protein expression. Statistical significance was defined as a P value less than 0.05. Analyses were performed using a statistical software package (SPSS version 16.0 for Windows; SPS, Inc; Chicago, IL, USA) and GraphPad software.

## Results

### ALDH2*2 KI Mice Are Susceptible to HS-Induced ALI *In Vivo*

**Figure 1:**
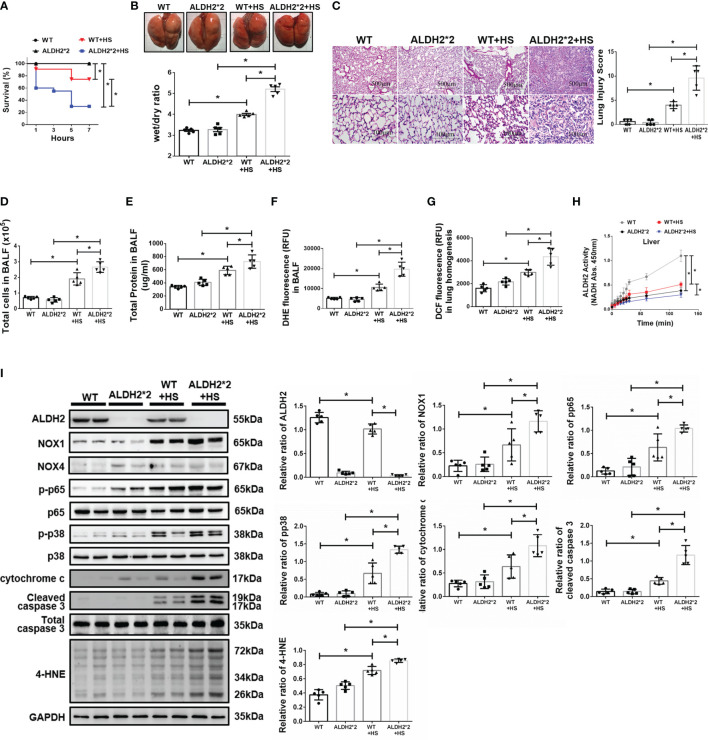
Effects of ALDH2 on HS-induced ALI *in vivo*. C57BL/6J WT and ALDH2*2 KI mice were exposed to WBH (42°C, 80% RH for 1 h) and then analyzed. **(A)** Survival of mice subjected to WBH (*n* = 20 in each group). **(B)** Representative images of the lungs and wet/dry ratio of the lungs (*n* = 5). **(C)** H&E stain of the lungs (*n* = 5). **(D)** Total cells in the BALF (*n* = 5). **(E)** Total protein in the BALF (*n* = 5). **(F)** ROS production in the BALF as determined by DHE fluorescence measurement using a fluorescence microplate reader with an excitation wavelength of 518 nm and an emission wavelength of 606 nm (*n* = 5). **(G)** ROS production in lung homogenates as determined by DCF fluorescence measurement using a fluorescence microplate reader with an excitation wavelength of 488 nm and an emission wavelength of 535 nm (*n* = 5). **(H)** The ALDH2 activity in liver homogenates was measured by NADH production using the O.D. absorbance at 450 nm in a microplate reader (*n* = 5). **(I)** The protein and 4-HNE levels in lung homogenates were measured by immunoblotting. Densitometric analysis was conducted with imaging processing software. The data were quantified by normalization to GAPDH; phosphorylated proteins were normalized to total proteins (*n* = 5). The data are expressed as the mean ± SD. Statistical significance is indicated as *p < 0.05.

There were no significant differences in body temperature between wild-type (WT) and ALDH2*2 KI mice after WBH (**Supplementary Figure 1**). Compared to WT mice, ALDH2*2 KI mice were more vulnerable to WBH with increased mortality rates (**Figure 1A**). ALDH2*2 mice were susceptible to WBH-induced ALI, there was a significant increase in the wet/dry ratio of lung tissue in WBH-induced mice (**Figure 1B**). WBH significantly induced pathological fluid accumulation and inflammatory cell infiltration in the lung (**Figure 1C**). Increased inflammatory cells, protein levels and ROS production were observed in the BALF of ALDH2*2 KI mice subjected to WBH compared with WT mice (**Figures 1D** **–F**). In lung homogenates, the ROS production was increased in ALDH2*2 KI mice subjected to WBH compared with that in WT mice (**Figure 1G**). ALDH2 activity was decreased in the livers of ALDH2*2 KI mice compared with WT mice (**Figure 1H**). WBH significantly induced the accumulation of 4-HNE, NOX1, the phosphorylated-p65:p65 ratio and the phosphorylated-p38:p38 ratio (**Figure 1I**). There were no significant differences in NOX4 expression between the groups. In addition, there were significantly elevated serum levels of AST, CK and BUN in ALDH2*2 KI mice subjected to WBH compared with WT mice (**Supplementary Figure 2**). Taken together, these results indicate that ALDH2*2 KI mice are susceptible to WBH-induced ALI.

### Silencing ALDH2 Exacerbates Heat Stress Induced Inflammatory Pathways and Reduced the Viability of HUVECs *In Vitro*

**Figure 2:**
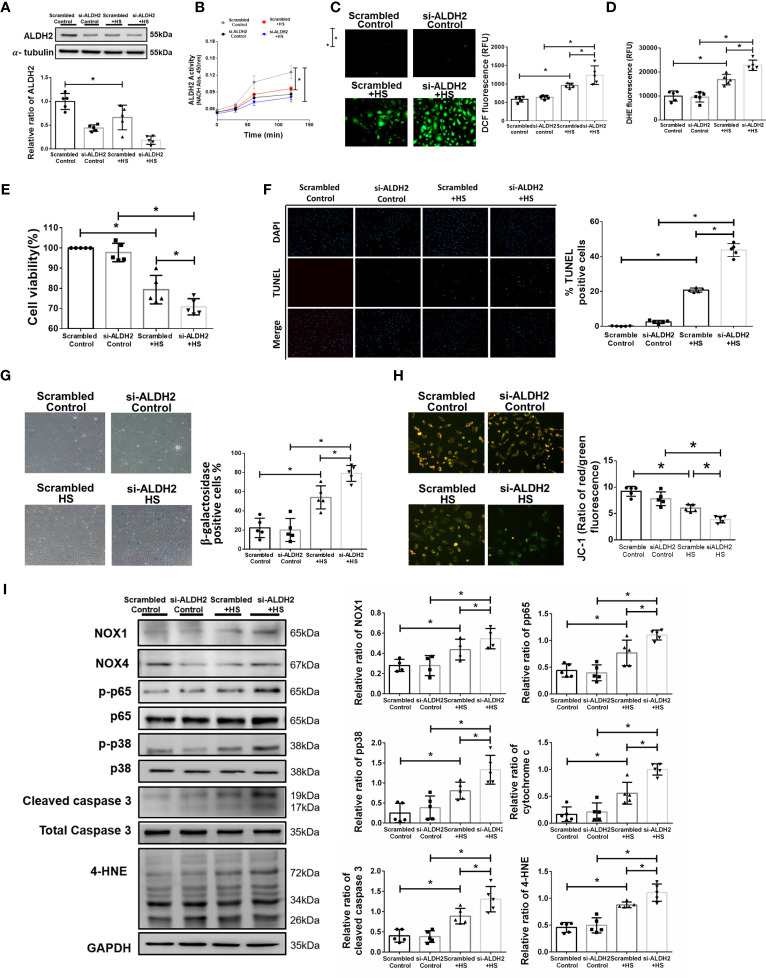
Silencing ALDH2 augmented heat stress-induced activation of NF-κB, ROS production and apoptosis in HUVECs *in vitro.* HUVECs were transfected with ALDH2 siRNA or control siRNA (scramble) before heat stress induction (42°C for 2 h). **(A)** ALDH2 protein expression was measured by immunoblotting (*n* = 5). **(B)** The ALDH2 activity in cell lysate was determined by measuring NADH production based on the O.D. absorbance at 450 nm in a microplate reader (*n* = 5). **(C)** Measurement of ROS production based on DCF fluorescence using a fluorescence microplate reader with an excitation wavelength of 488 nm and an emission wavelength of 535 nm (*n* = 5). **(D)** Measurement of cellular ROS production based on DHE fluorescence using a fluorescence microplate reader with an excitation wavelength of 518 nm and an emission wavelength of 606 nm (*n* = 5). **(E)** The viability of HUVECs was measured by the MTT assay based on the O.D. absorbance at 570 nm in a microplate reader (*n* = 5). **(F)** The levels of apoptosis were measured by the TUNEL assay as determined fluorescence microscopy. The percentage of apoptotic cells was determined based on the number of TUNEL-positive cells among the total number of cells (*n* = 5). **(G)** The levels of senescence were measured by β-galactosidase activity detection using bright field microscopy (*n* = 5). **(H)** Detection of mitochondrial dysfunction by the JC-1 assay, revealing a decrease in the mitochondrial membrane potential (ΔΨm) in live cells as determined by fluorescence microscopy and fluorescence microplate reader. The ΔΨm level is expressed as the merge of the red and green channels, and the data were quantified as the ratio of red fluorescence intensity to the green fluorescence intensity (*n* = 5). **(I)** The protein and 4-HNE levels in lung homogenates were measured by immunoblotting. Densitometric analysis was conducted with imaging processing software. The data were quantified by normalization to GAPDH; phosphorylated proteins were normalized to total proteins (*n* = 5). The data are expressed as the mean ± SD. Statistical significance is indicated as *p < 0.05.

To further confirm the effect of ALDH2 on heat stress *in vitro*, silencing ALDH2 was used in HUVECs with or without heat stress. As expected, the protein and activity of ALDH2 were decreased by silencing ALDH2 (**Figures 2A** **, B**). There was increased ROS accumulation in HUVECs subjected to 42°C for 2 h (**Figures 2C** **, D**). Silencing ALDH2 reduced the viability and exaggerated apoptosis and senescence of HUVECs subjected to heat stress (**Figures 2E** **–G** **)**. Consistent with the results of previous studies (5, 47), heat stress induced mitochondrial dysfunction and apoptosis. We found silencing ALDH2 attenuated the level of ΔΨm (**Figure 2H**) and increased the expression of cytochrome c and cleaved caspase 3 relative to the HS in HUVECs (**Figure 2I**). Meanwhile, the phosphorylated-p65:p65 ratio, the phosphorylated-p38:p38 ratio, the expression of NOX1 and the toxic 4-HNE were accumulated in the silencing ALDH2 subjected to HS in HUVECs (**Figure 2I**). NOX4 expression was not significantly different between the group. These results suggest that reduced ALDH2 exacerbates heat stress induced NF-kB inflammatory pathways, ROS production, mitochondrial dysfunction and reduces the viability of HUVECs *in vitro*.

### Alda-1 Attenuates Heat Stress-Induced Activation of Inflammatory Pathways and Preserved Viability in HUVECs *In Vitro*

**Figure 3:**
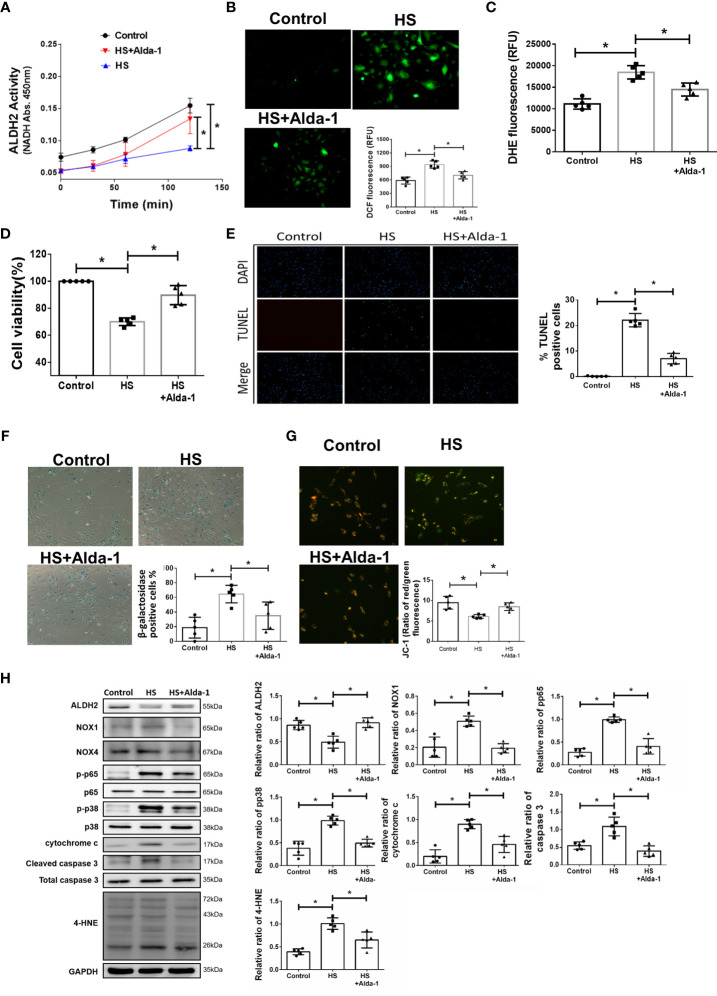
Effects of Alda-1 on HS-induced vascular inflammation, ROS and apoptosis *in vitro*. Alda-1 (20 μM) was added to HUVECs for 6 h before HS (42°C for 2 h). **(A)** The ALDH2 activity in cell lysate was determined by measuring NADH production based on the O.D. absorbance at 450 nm in a microplate reader (*n* = 5). **(B)** Measurement of ROS production based on DCF fluorescence as determined by using a fluorescence microplate reader with an excitation wavelength of 488 nm and an emission wavelength of 535 nm (*n* = 5). **(C)** Measurement of cellular ROS production based on DHE fluorescence as determined using a fluorescence microplate reader with an excitation wavelength of 518 nm and an emission wavelength of 606 nm (*n* = 5). **(D)** The viability of HUVECs was measured by the MTT assay based on the O.D. absorbance at 570 nm in a microplate reader (*n* = 5). **(E)** The levels of apoptosis were measured by the TUNEL assay as determined by fluorescence microscopy. The percentage of apoptotic cells was determined based on the number of TUNEL-positive cells among the total number of cells. **(F)** The levels of senescence were measured by β-galactosidase activity detection using bright field microscopy (*n* = 5). **(G)** Detection of mitochondrial dysfunction by the JC-1 assay, revealing that the mitochondrial membrane potential (ΔΨm) was decreased in live cells as determined by fluorescence microscopy and a fluorescence microplate reader. The ΔΨm level is expressed as the merge of the red and green channels, and the data were quantified as the ratio of red fluorescence intensity to green fluorescence intensity (*n* = 5). **(H)** The protein and 4-HNE levels in lung homogenates were measured by immunoblotting. Densitometric analysis was conducted with imaging processing software. The data were quantified by normalization to GAPDH; phosphorylated proteins were normalized to total proteins (*n* = 5). The data are expressed as the mean ± SD. Statistical significance is indicated as *p < 0.05.

To understand the effect of ALDH2 activation on HS, Alda-1 was pretreated in HS induced HUVECs. Consistent with previous studies (29, 31, 48), Alda-1 (20μM) significantly augmented ALDH2 activity (**Figure 3A**) and reduced heat stress-induced ROS accumulation (**Figures 3B, C** **)**. Alda-1 ameliorated the heat stress-induced cell death (**Figure 3D** **)** and reduced heat stress-induced apoptosis (**Figure 3E** **)** and senescence (**Figure 3F** **)** in HUVECs. Alda-1 reversed the heat stress-reduced ΔΨm (**Figure 3G** **).** Alda-1 attenuated the heat stress-induced phosphorylated-p65:p65 ratio, phosphorylated-p38:p38 ratio, cytochrome c, cleaved caspase 3, NOX1 and 4-HNE accumulation (**Figure 3H**). There were no significant differences regarding NOX4 expression. These results suggest that activation of ALDH2 by Alda-1 reduces heat stress induced mitochondrial dysfunction and ROS accumulation of HUVECs *in vitro*.

### Alda-1 Ameliorates WBH-Induced ALI *In Vivo*

**Figure 4:**
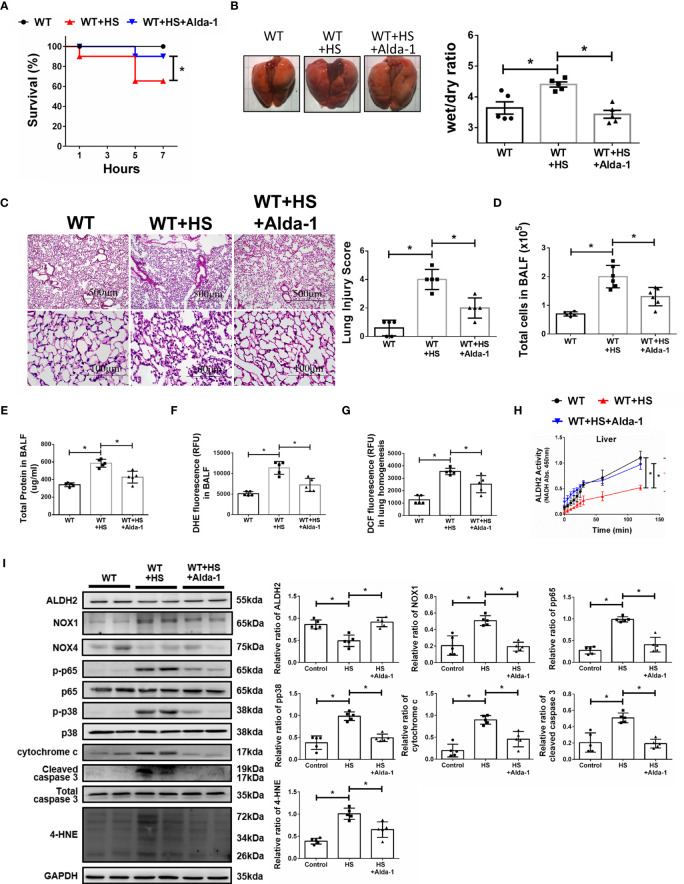
Alda-1 attenuates HS-induced ALI *in vivo.* Alda-1-pretreated C57BL/6J mice were exposed to WBH (42°C, 80% RH for 1 h) and then analyzed. **(A)** Survival of mice subjected to WBH (*n* = 20 in each group). **(B)** Representative images of the lungs and wet/dry ratio of the lungs (*n* = 5). **(C)** H&E stain of the lungs (*n* = 5). **(D)** Total cells in the BALF (*n* = 5). **(E)** Total protein in the BALF (*n* = 5). **(F)** Measurement of ROS production in BALF based on DHE fluorescence as determined using a fluorescence microplate reader with an excitation wavelength of 518 nm and an emission wavelength of 606 nm (*n* = 5). **(G)** Measurement of ROS production in lung homogenates based on DCF fluorescence as determined using a fluorescence microplate reader with an excitation wavelength of 488 nm and an emission wavelength of 535 nm (*n* = 5). **(H)** The ALDH2 activity in lung homogenates was measured by NADH production based on the O.D. absorbance at 450 nm in a microplate reader (*n* = 5). **(I)** The protein and 4-HNE levels in lung homogenates were measured by immunoblotting. Densitometric analysis was conducted with imaging processing software. The data were quantified by normalization to GAPDH; phosphorylated proteins were normalized to total proteins (*n* = 5). The data are expressed as the mean ± SD. Statistical significance is indicated as *p < 0.05.

We then tested the protective effects of Alda-1 on WBH-induced ALI *in vivo*. Pretreatment with Alda-1 significantly increased HS-related survival rates by 25% (**Figure 4A**). Alda-1 ameliorated WBH-induced ALI, there was a significant decrease in wet/dry ratio of lung tissue in mice that received WBH exposure with Alda-1 pretreatment (**Figure 4B**). In addition, Alda-1 significantly reduced pathological fluid accumulation and inflammatory cell infiltration in the lung (**Figure 4C**). There were decreased inflammatory cell protein levels and ROS production in the BALF of Alda-1-pretreated mice subjected to WBH (**Figures 4D** **–F** **)**. In lung homogenates, mice subjected to WBH increased ROS accumulation by 2.8-fold relative to the control, whereas pretreatment with Alda-1 reduced the level by 2.9-fold (**Figure 4G** **)**. As expected, Alda-1-treated mice had significantly elevated ALDH2 activity (**Figure 4H**) in liver homogenates. Alda-1 significantly decreased the WBH-induced accumulation of 4-HNE, NOX1 expression, the phosphorylated-p65:p65 ratio, the phosphorylated-p38:p38 ratio, cytochrome c expression and cleaved caspase 3 expression (**Figure 4I**). NOX4 expression was not significantly different between the groups. Alda-1 attenuated HS-induced elevations in serum levels of AST, CK, and BUN (**Supplementary Figure 2**). These results suggest that pretreatment with Alda-1 ameliorates WBH-induced ALI *in vivo* through reduced activation of NF-kB and apoptotic pathways and ROS accumulation.

## Discussion

**Figure 5:**
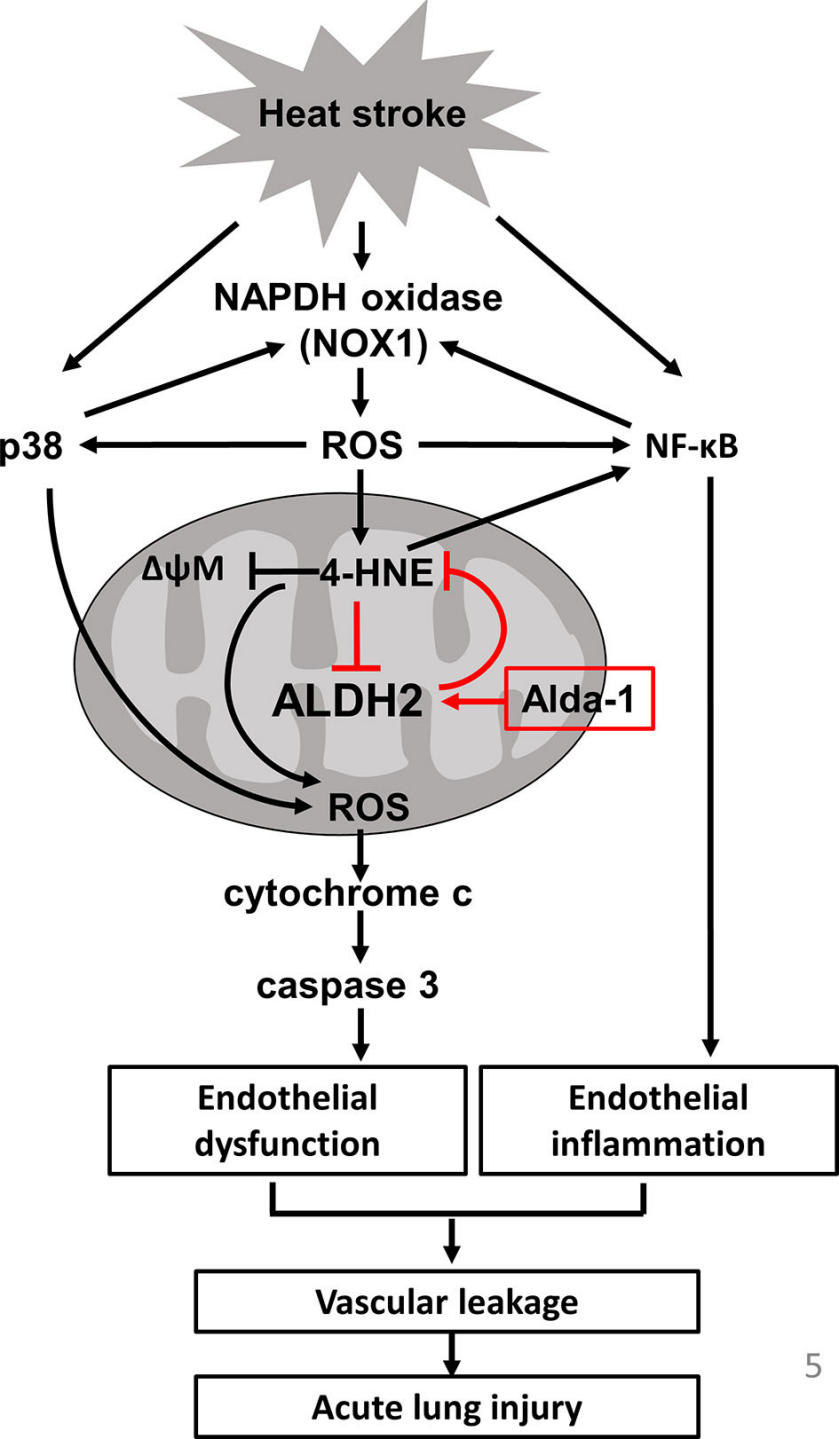
Schematic of the role of ALDH2 in HS.

In this study, we demonstrated the protective role of ALDH2 in HS-induced ALI. Alda-1 attenuated HS-induced ALI by reducing the accumulation of ROS and toxic aldehydes and alleviating vascular inflammation and endothelial dysfunction. A schematic is shown in **Figure 5**.

In this study, we highlight 4-HNE and ROS production as a vicious cycle in heatstroke-induced ALI and the roles of ALDH2 in breaking the vicious cycle. Activation of NF-κB-induced NOXs activation and ROS production (34, 49, 50). Previous studies indicated that 4-HNE increased the production of ROS through NOX and 5-lipoxygenase (5-LO) (51). 5-LO expression induced by HNE is regulated by activation of the p38 MAPK and NF-κB pathways in macrophages (52). The activation of 5-LO by HNE enhanced the CD36 expression and MMP-2 production and led to macrophage foam cell formation and atherosclerotic plaque instability (53, 54). Nicotinamide adenine dinucleotide phosphate oxidases (NOX) are transmembrane enzymes that catalyze the generation of superoxide anions through the transfer of electrons from NADPH to molecular oxygen and NOXs-derived ROS induce endothelial dysfunction (55). NOX1 is a major source of ROS that induces p38 and NF-kB activation and 4-HNE expression, thereby causing inflammation and oxidative stress (56, 57). In addition, NF-kB and p38 activation upregulate NOX-1 overexpression in ECs (38, 58). NOX-derived ROS causes a decrease in the ΔΨm, resulting in an increase in mitochondrial-derived ROS, whereas mitochondrial ROS production cause a secondary activation of NOXs (59). NOX-derived ROS are mediators of endogenous biological changes under HS (60), consistently, we also found that acute HS induces NOX1 overexpression but not NOX4. Using mice carrying the human ALDH2*2 dysfunctional polymorphism, we demonstrated that this ALDH2*2 variant conferred susceptibility to HS, as evidenced by increased ROS and 4-HNE accumulation, vascular inflammation and endothelial dysfunction. Previous studies have shown increased inflammatory markers in patients with HS and in animal models of HS. HS resembles sepsis in several aspects, and increasing evidence suggests that endotoxemia and cytokines may be implicated in HS pathogenesis (16). HS significantly elevates the levels of cytokines in BALF and activates the NF-κB signaling pathway in lung tissue (61). HS induces p38 activation and inflammatory signaling, apoptosis and pyroptosis in vascular cells (14, 15, 17, 62). Consistent with previous studies, we also found that mice subjected to HS had increased accumulation of ROS and 4-HNE (18). ALDH2*2 variants are associated with the increased incidence of several neurodegenerative, cardiovascular and endocrinological diseases as well as lung and alimentary tract cancers (62). The accumulation of 4-HNE has been implicated in the pathogenesis of numerous oxidative stress-related diseases and in the development and progression of CVDs (63–65). The rs671 polymorphism in ALDH2 promotes macrophage foam cell formation and vascular inflammation in atherosclerosis (65, 66). Patients with ALDH2 deficiency have higher postoperative oxidative stress levels and are susceptible to cisplatin-induced cytotoxicity *via* the overproduction of ROS (67, 68). Genetic ALDH2-deficient mice are prone to ethanol-induced liver inflammation and fibrosis by paracrine activation of IL-6 in Kupffer cells (69). Consistent with previous studies, we also found that decreased ALDH2 activity resulted in enhanced phosphorylation of p65 and p38 and apoptosis in ECs (40, 70, 71).

The primary treatment for HS is the alleviation of hyperthermia. Adjunctive therapies for organ injury are still limited (3). We provided a rationale for the use of an ALDH2 activator as an adjunctive HS treatment in this study. ALDH2 was shown to protect against oxidative stress and the subsequent accumulation of toxic aldehydes and adducts in IRI (24). ALDH2 protects against heat shock and is involved in the pathogenesis of sepsis (72). ALDH2 overexpression prevented acetaldehyde-induced cell injury and decreased apoptosis in ECs and oxidative stress-induced endothelial dysfunction (73–75). Alda-1 binds to ALDH2 and restores ALDH activity by acting as a structural chaperone (29). Previous studies demonstrated decreased ALDH2 expression due to increased ALDH2 protein turnover in both humans with the ALDH2*2 variant and in ALDH2*2 KI mice (76, 77). Aldehydes and 4-HNE also inactivate ALDH2 itself and the mitochondrial electron transport chain (64). Alda-1 restored the high-glucose-induced decrease in ALDH2 protein expression and activity in rat cardiomyocytes (78). Consistently, we also found that Alda-1 increased ALDH2 activities and protein expression under heat stress. Nonetheless, Alda-1 increased the ALDH2 activity but not its protein expression in the control cells. We speculated that Alda-1 stabilizes heat stress-induced ALDH2 degradation by acting as a chemical chaperone (29). ALDH2 protects against angiotensin II-induced ROS generation and prevents ROS-induced vessel contraction (28). Pretreatment with Alda-1 upregulated ALDH2 activity and reduced 4-HNE and MDA accumulation in various models of intestinal IRI (32). Accelerated aldehyde degradation by Alda-1 also decreased bile duct ligation-induced liver necrosis, inflammation and fibrosis (33). Alda-1 attenuated 4-HNE-induced vascular smooth muscle cell proliferation and migration by regulating NF-κB activation, ameliorated vascular remodeling in a mouse model of pulmonary hypertension and inhibited atherosclerosis and fatty liver in hyperlipidemic mice (30, 31). Alda-1 inhibits oxidized low-density lipoprotein-induced priming and activation of the NLRP3 inflammasome by reducing oxidative stress in macrophages (79). Alda-1 attenuated high-glucose-induced mitochondrial injury in H9c2 cells (80). In this study, we found that Alda-1 attenuated HS-induced accumulation of 4-HNE and ROS and further prevented HS-induced ALI. Our previous study also demonstrated that Alda-1 attenuated AngII-induced abdominal aortic aneurysm (AAA) in ApoE-KO mice (34).

Increased immune cells in BALF could be due to endothelial activation/dysfunction and recruitment of inflammatory cells. Consistent with previous studies (14, 17), We have found that there were increased total cells in the BALF in mice subjected to HS. A recent study regarding hyperoxia induced ALI also revealed that pretreatment with Alda-1 reduced hyperoxia induced immune cell infiltration, alveolar damage and lung inflammation and preserved alveolar permeability through the activation of Akt and mTOR pathways (35). Here we demonstrated that pretreatment with Alda-1 prevented HS-induced ALI by reducing ROS production, toxic aldehydes and mitochondrial injury and preserving the viability of ECs.

Through HS clearly induces endothelial barrier dysfunction and hyperpermeability (13–17), the potential role of hydrostatic lung edema formation in HS induced ALI models are otherwise limited. Accumulated ROS also induced lung edema formation through downregulation of alveolar epithelial Na/K-ATPase activity with impaired alveolar fluid reabsorption (81).

### Limitations

We are aware that the development of HS could be multifactorial in nature, including other cell types, mediators and pathways. Infiltrated immune cells in BALF could be determined to further elucidate the roles of ALDH2 on HS. The roles of ALDH2 in other inflammatory cells as well HS-induced changes in pulmonary hemodynamics, lung endothelial permeability and the alveolar fluid resorption should be further investigated. Heat shock response systems, including the heat shock factor-1 (HSF-1) and heat shock protein (HSP) stress systems, provide protection against thermal insult by regulating the transcription of several HSPs and promoting chaperone activities to alleviate proteotoxic stresses in eukaryotic cells. The interplay among HSF-1, HSPs and ALDH2 should be further explored. HSF-1 can upregulate the expression of ALDH2 *via* protein kinase C (82). 4-HNE targets and impairs the function of HSP70 and the endoplasmic reticulum homolog of HSP70, glucose-regulated protein 78 (83, 84).

## Conclusion

We demonstrated the crucial role of ALDH2 in protecting against heat stress-induced ROS production and vascular inflammation and preserving the viability of ECs. ALDH2 activation by Alda-1 attenuated WBH-induced ALI *in vivo*.

## Data Availability Statement

The original contributions presented in the study are included in the article/**Supplementary Material**. Further inquiries can be directed to the corresponding authors.

## Ethics Statement

The animal study was reviewed and approved by Tri-Service General Hospital, National Defense Medical Center, Taipei, Taiwan.

## Author Contributions

S-HT and Y-JH participated in the design and planning of the study. H-YT, W-CH, and C-YL performed and analyzed experiments. J-CW wrote the article. L-AH, Y-HY, and PC prepared the figures. All authors have read, edited, and approved the manuscript.

## Funding

This study was supported by grants from Tri-Service General Hospital, National Defense Medical Center, Taipei, Taiwan (TSGH-D-109071, TSGH-D-109125, TSGH-E-110222 and TSGH-E-110223), the Ministry of National Defense-Medical Affairs Bureau (MAB-108-018 and MAB-110-121) and the Ministry of Science and Technology (MOST 108-2314-B-016-047-MY3 and MOST 110-2314-B-016-054).

## Conflict of Interest

The authors declare that the research was conducted in the absence of any commercial or financial relationships that could be construed as a potential conflict of interest.

## Publisher’s Note

All claims expressed in this article are solely those of the authors and do not necessarily represent those of their affiliated organizations, or those of the publisher, the editors and the reviewers. Any product that may be evaluated in this article, or claim that may be made by its manufacturer, is not guaranteed or endorsed by the publisher.

## Supplementary Material

Supplementary Figure 1 The core temperature of the experimental animals. Body temperature changes in WT and ALDH2*2 KI mice subjected to WBH.

Supplementary Figure 2 The serum levels of AST, CK, and BUN in experimental animals. ALDH2*2 KI mice and their littermates **(A)** and Alda-1-treated mice **(B)** subjected to WBH. The data are expressed as the mean ± SD. Statistical significance is indicated as **p* < 0.05.

Supplementary Figure 3 Alda-1 increased the ALDH2 activity but not its expression in HUVECs. **(A)** ALDH2 protein expression was measured by immunoblotting (*n* = 5). **(B)** ALDH2 activity was measured by NADH production as determined by the O.D. absorbance at 450 nm in a microplate reader (*n* = 5). The data are expressed as the mean ± SD. Statistical significance is indicated as **p* < 0.05.

## References

[1] Sankoff J. Heat Illnesses: A Hot Topic in the Setting of Global Climate Change. Aust Fam Physician (2015) 44(1-2):22–6. 25688955

[2] Hess JJ Saha S Luber G. Summertime Acute Heat Illness in U.S. Emergency Departments From 2006 Through 2010: Analysis of a Nationally Representative Sample. Environ Health Perspect (2014) 122(11):1209–15. doi: 10.1289/ehp.1306796 PMC4216158 24937159

[3] Epstein Y Yanovich R. Heatstroke. N Engl J Med (2019) 380(25):2449–59. doi: 10.1056/NEJMra1810762 31216400

[4] Leon LR Bouchama A. Heat Stroke. Compr Physiol (2015) 5(2):611–47. doi: 10.1002/cphy.c140017 25880507

[5] Ganesan S Volodina O Pearce SC Gabler NK Baumgard LH Rhoads RP. Acute Heat Stress Activated Inflammatory Signaling in Porcine Oxidative Skeletal Muscle. Physiol Rep (2017) 5(16):e13397–406. doi: 10.14814/phy2.13397 PMC5582270 28830980

[6] Stallings JD Ippolito DL Rakesh V Baer CE Dennis WE Helwig BG. Patterns of Gene Expression Associated With Recovery and Injury in Heat-Stressed Rats. BMC Genomics (2014) 15(1):1058. doi: 10.1186/1471-2164-15-1058 25471284 PMC4302131

[7] Chauhan NR Kapoor M Prabha Singh L Gupta RK Chand Meena R Tulsawani R. Heat Stress-Induced Neuroinflammation and Aberration in Monoamine Levels in Hypothalamus Are Associated With Temperature Dysregulation. Neuroscience (2017) 358:79–92. doi: 10.1016/j.neuroscience.2017.06.023 28663093

[8] Wang C Fan F Cao Q Shen C Zhu H Wang P. Mitochondrial Aldehyde Dehydrogenase 2 Deficiency Aggravates Energy Metabolism Disturbance and Diastolic Dysfunction in Diabetic Mice. J Mol Med (Berl) (2016) 94(11):1229–40. doi: 10.1007/s00109-016-1449-5 27488451

[9] Zhang M Zhu X Tong H Lou A Li Y Li Y. AVE 0991 Attenuates Pyroptosis and Liver Damage After Heatstroke by Inhibiting the ROS-NLRP3 Inflammatory Signalling Pathway. BioMed Res Int (2019) 2019:1806234. doi: 10.1155/2019/1806234 31531346 PMC6720052

[10] Lawrence T. The Nuclear Factor NF-KappaB Pathway in Inflammation. Cold Spring Harb Perspect Biol (2009) 1(6):a001651. doi: 10.1101/cshperspect.a001651 20457564 PMC2882124

[11] Park S Lim Y Lee D Elvira R Lee JM Lee MR. Modulation of Protein Synthesis by Eif2alpha Phosphorylation Protects Cell From Heat Stress-Mediated Apoptosis. Cells (2018) 7(12):1–15. doi: 10.3390/cells7120254 PMC6316477 30544621

[12] Selkirk GA McLellan TM Wright HE Rhind SG. Mild Endotoxemia, NF-kappaB Translocation, and Cytokine Increase During Exertional Heat Stress in Trained and Untrained Individuals. Am J Physiol Regul Integr Comp Physiol (2008) 295(2):R611–23. doi: 10.1152/ajpregu.00917.2007 18565834

[13] Mundhara N Majumder A Panda D. Hyperthermia Induced Disruption of Mechanical Balance Leads to G1 Arrest and Senescence in Cells. Biochem J (2020) 478(1):179–96. doi: 10.1042/bcj20200705 33346336

[14] Chen Y Tong H Pan Z Jiang D Zhang X Qiu J. Xuebijing Injection Attenuates Pulmonary Injury by Reducing Oxidative Stress and Proinflammatory Damage in Rats With Heat Stroke. Exp Ther Med (2017) 13(6):3408–16. doi: 10.3892/etm.2017.4444 PMC5450780 28588676

[15] Pei Y Geng Y Su L. Pyroptosis of HUVECs can be Induced by Heat Stroke. Biochem Biophys Res Commun (2018) 506(3):626–31. doi: 10.1016/j.bbrc.2018.10.051 30454698

[16] Lu KC Wang JY Lin SH Chu P Lin YF. Role of Circulating Cytokines and Chemokines in Exertional Heatstroke. Crit Care Med (2004) 32(2):399–403. doi: 10.1097/01.ccm.0000108884.74110.d9 14758154

[17] Zhou G Chen Z Li J Guo X Qin K Luo J. Role of the Receptor for Advanced Glycation End Products in Heat Stress-Induced Endothelial Hyperpermeability in Acute Lung Injury. Front Physiol (2020) 11:1087. doi: 10.3389/fphys.2020.01087 33192536 PMC7643755

[18] Tao Z Cheng M Wang SC Lv W Hu HQ Li CF. JAK2/STAT3 Pathway Mediating Inflammatory Responses in Heatstroke-Induced Rats. Int J Clin Exp Pathol (2015) 8(6):6732–9. PMC4525890 26261556

[19] Liu Z Chen J Hu L Li M Liang M Chen J. Expression Profiles of Genes Associated With Inflammatory Responses and Oxidative Stress in Lung After Heat Stroke. Biosci Rep (2020) 40(6):BSR2019048–62. doi: 10.1042/bsr20192048 PMC7276522 32436952

[20] Li L Tan H Zou Z Gong J Zhou J Peng N. Preventing Necroptosis by Scavenging ROS Production Alleviates Heat Stress-Induced Intestinal Injury. Int J Hyperthermia (2020) 37(1):517–30. doi: 10.1080/02656736.2020.1763483 32423248

[21] Chen C-H Ferreira JCB Gross ER Mochly-Rosen D. Targeting Aldehyde Dehydrogenase 2: New Therapeutic Opportunities. Physiol Rev (2014) 94(1):1–34. doi: 10.1152/physrev.00017.2013 24382882 PMC3929114

[22] Zhang Y Ren J. ALDH2 in Alcoholic Heart Diseases: Molecular Mechanism and Clinical Implications. Pharmacol Ther (2011) 132(1):86–95. doi: 10.1016/j.pharmthera.2011.05.008 21664374 PMC3144032

[23] Zhang Y Babcock SA Hu N Maris JR Wang H Ren J. Mitochondrial Aldehyde Dehydrogenase (ALDH2) Protects Against Streptozotocin-Induced Diabetic Cardiomyopathy: Role of GSK3beta and Mitochondrial Function. BMC Med (2012) 10:40. doi: 10.1186/1741-7015-10-40 22524197 PMC3439670

[24] Panisello-Rosello A Lopez A Folch-Puy E Carbonell T Rolo A Palmeira C. Role of Aldehyde Dehydrogenase 2 in Ischemia Reperfusion Injury: An Update. World J Gastroenterol (2018) 24(27):2984–94. doi: 10.3748/wjg.v24.i27.2984 PMC6054945 30038465

[25] Hao P-P Chen Y-G Wang J-L Wang XL Zhang Y. Meta-Analysis of Aldehyde Dehydrogenase 2 Gene Polymorphism and Alzheimer's Disease in East Asians. Can J Neurol Sci Le J Canadien Des Sci Neurologiques (2011) 38(3):500–6. doi: 10.1017/s0317167100011938 21515512

[26] Schneider C Porter NA Brash AR. Routes to 4-Hydroxynonenal: Fundamental Issues in the Mechanisms of Lipid Peroxidation. J Biol Chem (2008) 283(23):15539–43. doi: 10.1074/jbc.R800001200 PMC2414272 18285327

[27] Mali VR Palaniyandi SS. Regulation and Therapeutic Strategies of 4-Hydroxy-2-Nonenal Metabolism in Heart Disease. Free Radical Res (2014) 48(3):251–63. doi: 10.3109/10715762.2013.864761 24237196

[28] Choi H Tostes RC Webb RC. Mitochondrial Aldehyde Dehydrogenase Prevents ROS-Induced Vascular Contraction in Angiotensin-II Hypertensive Mice. J Am Soc Hypertens (2011) 5(3):154–60. doi: 10.1016/j.jash.2011.02.005 PMC3085594 21459068

[29] Perez-Miller S Younus H Vanam R Chen CH Mochly-Rosen D Hurley TD. Alda-1 Is an Agonist and Chemical Chaperone for the Common Human Aldehyde Dehydrogenase 2 Variant. Nat Struct Mol Biol (2010) 17(2):159–64. doi: 10.1038/nsmb.1737 PMC2857674 20062057

[30] Xu T Liu S Ma T Jia Z Zhang Z Wang A. Aldehyde Dehydrogenase 2 Protects Against Oxidative Stress Associated With Pulmonary Arterial Hypertension. Redox Biol (2017) 11:286–96. doi: 10.1016/j.redox.2016.12.019 PMC5192477 28030785

[31] Stachowicz A Olszanecki R Suski M Wisniewska A Toton-Zuranska J Madej J. Mitochondrial Aldehyde Dehydrogenase Activation by Alda-1 Inhibits Atherosclerosis and Attenuates Hepatic Steatosis in Apolipoprotein E-Knockout Mice. J Am Heart Assoc (2014) 3(6):e001329. doi: 10.1161/jaha.114.001329 25392542 PMC4338726

[32] Zhu Q He G Wang J Wang Y Chen W. Pretreatment With the ALDH2 Agonist Alda-1 Reduces Intestinal Injury Induced by Ischaemia and Reperfusion in Mice. Clin Sci (Lond) (2017) 131(11):1123–36. doi: 10.1042/cs20170074 PMC5434792 28325855

[33] Wimborne HJ Takemoto K Woster PM Rockey DC Lemasters JJ Zhong Z. Aldehyde Dehydrogenase-2 Activation by Alda-1 Decreases Necrosis and Fibrosis After Bile Duct Ligation in Mice. Free Radic Biol Med (2019) 145:136–45. doi: 10.1016/j.freeradbiomed.2019.09.026 PMC6880805 31557514

[34] Tsai SH Hsu LA Tsai HY Yeh YH Lu CY Chen PC. Aldehyde Dehydrogenase 2 Protects Against Abdominal Aortic Aneurysm Formation by Reducing Reactive Oxygen Species, Vascular Inflammation, and Apoptosis of Vascular Smooth Muscle Cells. FASEB J (2020) 34(7):9498–511. doi: 10.1096/fj.201902550RRR 32463165

[35] Sidramagowda Patil S Hernández-Cuervo H Fukumoto J Krishnamurthy S Lin M Alleyn M. Alda-1 Attenuates Hyperoxia-Induced Acute Lung Injury in Mice. Front Pharmacol (2020) 11:597942. doi: 10.3389/fphar.2020.597942 33597876 PMC7883597

[36] Lu Q Mundy M Chambers E Lange T Newton J Borgas D. Alda-1 Protects Against Acrolein-Induced Acute Lung Injury and Endothelial Barrier Dysfunction. Am J Respir Cell Mol Biol (2017) 57(6):662–73. doi: 10.1165/rcmb.2016-0342OC PMC5765414 28763253

[37] Patil SS Hernández-Cuervo H Fukumoto J Narala VR Saji S Borra M. Alda-1 Attenuates Hyperoxia-Induced Mitochondrial Dysfunction in Lung Vascular Endothelial Cells. Aging (Albany NY) (2019) 11(12):3909–18. doi: 10.18632/aging.102012 PMC6628993 31209184

[38] Huang J Li L Yuan W Zheng L Guo Z Huang W. NEMO-Binding Domain Peptide Attenuates Lipopolysaccharide-Induced Acute Lung Injury by Inhibiting the NF-κb Signaling Pathway. Mediators Inflamm (2016) 2016:7349603. doi: 10.1155/2016/7349603 27956761 PMC5120201

[39] Chen C-H Budas GR Churchill EN Disatnik M-H Hurley TD Mochly-Rosen D. Activation of Aldehyde Dehydrogenase-2 Reduces Ischemic Damage to the Heart. Sci (New York NY) (2008) 321(5895):1493–5. doi: 10.1126/science.1158554 PMC2741612 18787169

[40] Lin CY Hsu CC Lin MT Chen SH. Flutamide, an Androgen Receptor Antagonist, Improves Heatstroke Outcomes in Mice. Eur J Pharmacol (2012) 688(1-3):62–7. doi: 10.1016/j.ejphar.2012.05.002 22609231

[41] Sun L Ferreira JC Mochly-Rosen D. ALDH2 Activator Inhibits Increased Myocardial Infarction Injury by Nitroglycerin Tolerance. Sci Transl Med (2011) 3(107):107ra11. doi: 10.1126/scitranslmed.3002067 PMC3547591 22049071

[42] Pan G Roy B Palaniyandi SS. Diabetic Aldehyde Dehydrogenase 2 Mutant (ALDH2*2) Mice Are More Susceptible to Cardiac Ischemic-Reperfusion Injury Due to 4-Hydroxy-2-Nonenal Induced Coronary Endothelial Cell Damage. J Am Heart Assoc (2021) 10(18):e021140. doi: 10.1161/jaha.121.021140 34482710 PMC8649540

[43] National Research Council Committee for the Update of the Guide for the C, Use of Laboratory A. The National Academies Collection: Reports Funded by National Institutes of Health. In: Guide for the Care and Use of Laboratory Animals. Washington (DC: National Academies Press (US) Copyright © 2011, National Academy of Sciences (2011).

[44] Tang SE Wu CP Wu SY Peng CK Perng WC Kang BH. Stanniocalcin-1 Ameliorates Lipopolysaccharide-Induced Pulmonary Oxidative Stress, Inflammation, and Apoptosis in Mice. Free Radic Biol Med (2014) 71:321–31. doi: 10.1016/j.freeradbiomed.2014.03.034 24685991

[45] Lin HJ Wu CP Peng CK Lin SH Uchida S Yang SS. With-No-Lysine Kinase 4 Mediates Alveolar Fluid Regulation in Hyperoxia-Induced Lung Injury. Crit Care Med (2015) 43(10):e412–9. doi: 10.1097/ccm.0000000000001144 26035408

[46] Chou SH Lan J Esposito E Ning M Balaj L Ji X. Extracellular Mitochondria in Cerebrospinal Fluid and Neurological Recovery After Subarachnoid Hemorrhage. Stroke (2017) 48(8):2231–7. doi: 10.1161/strokeaha.117.017758 PMC5526718 28663512

[47] Gu ZT Wang H Li L Liu YS Deng XB Huo SF. Heat Stress Induces Apoptosis Through Transcription-Independent P53-Mediated Mitochondrial Pathways in Human Umbilical Vein Endothelial Cell. Sci Rep (2014) 4:4469. doi: 10.1038/srep04469 24667845 PMC3966036

[48] Zhang R Liu B Fan X Wang W Xu T Wei S. Aldehyde Dehydrogenase 2 Protects Against Post-Cardiac Arrest Myocardial Dysfunction Through a Novel Mechanism of Suppressing Mitochondrial Reactive Oxygen Species Production. Front Pharmacol (2020) 11:373. doi: 10.3389/fphar.2020.00373 32292348 PMC7118728

[49] Echizen K Horiuchi K Aoki Y Yamada Y Minamoto T Oshima H. NF-κb-Induced NOX1 Activation Promotes Gastric Tumorigenesis Through the Expansion of SOX2-Positive Epithelial Cells. Oncogene (2019) 38(22):4250–63. doi: 10.1038/s41388-019-0702-0 PMC6756228 30700829

[50] Tsai SH Wang JC Liao WI Hsu YJ Lin CY Liao MT. Fucoidan Attenuates Angiotensin II-Induced Abdominal Aortic Aneurysms Through the Inhibition of C-Jun N-Terminal Kinase and Nuclear Factor κb Activation. J Vasc Surg (2018) 68(6s):72S–81S.e1. doi: 10.1016/j.jvs.2017.09.042 29290496

[51] Yun MR Park HM Seo KW Lee SJ Im DS Kim CD. 5-Lipoxygenase Plays an Essential Role in 4-HNE-Enhanced ROS Production in Murine Macrophages *via* Activation of NADPH Oxidase. Free Radic Res (2010) 44(7):742–50. doi: 10.3109/10715761003758122 20370567

[52] Lee SJ Kim CE Seo KW Kim CD. HNE-Induced 5-LO Expression Is Regulated by NF-{Kappa}B/ERK and Sp1/p38 MAPK Pathways *via* EGF Receptor in Murine Macrophages. Cardiovasc Res (2010) 88(2):352–9. doi: 10.1093/cvr/cvq194 20554538

[53] Seo KW Lee SJ Kim CE Yun MR Park HM Yun JW. Participation of 5-Lipoxygenase-Derived LTB(4) in 4-Hydroxynonenal-Enhanced MMP-2 Production in Vascular Smooth Muscle Cells. Atherosclerosis (2010) 208(1):56–61. doi: 10.1016/j.atherosclerosis.2009.06.012 19586628

[54] Yun MR Im DS Lee SJ Park HM Bae SS Lee WS. 4-Hydroxynonenal Enhances CD36 Expression on Murine Macrophages *via* P38 MAPK-Mediated Activation of 5-Lipoxygenase. Free Radic Biol Med (2009) 46(5):692–8. doi: 10.1016/j.freeradbiomed.2008.12.013 19135147

[55] Wenzel P Mollnau H Oelze M Schulz E Wickramanayake JM Müller J. First Evidence for a Crosstalk Between Mitochondrial and NADPH Oxidase-Derived Reactive Oxygen Species in Nitroglycerin-Triggered Vascular Dysfunction. Antioxid Redox Signal (2008) 10(8):1435–47. doi: 10.1089/ars.2007.1969 18522491

[56] Ho CC Chen YC Tsai MH Tsai HT Weng CY Yet SF. Ambient Particulate Matter Induces Vascular Smooth Muscle Cell Phenotypic Changes *via* NOX1/ROS/NF-κb Dependent and Independent Pathways: Protective Effects of Polyphenols. Antioxid (Basel) (2021) 10(5):742–50. doi: 10.3390/antiox10050782 PMC8156007 34069133

[57] Choi H Dikalova A Stark RJ Lamb FS. C-Jun N-Terminal Kinase Attenuates Tnfα Signaling by Reducing Nox1-Dependent Endosomal ROS Production in Vascular Smooth Muscle Cells. Free Radic Biol Med (2015) 86:219–27. doi: 10.1016/j.freeradbiomed.2015.05.015 26001727

[58] Song W Wei L Du Y Wang Y Jiang S. Protective Effect of Ginsenoside Metabolite Compound K Against Diabetic Nephropathy by Inhibiting NLRP3 Inflammasome Activation and NF-κb/P38 Signaling Pathway in High-Fat Diet/Streptozotocin-Induced Diabetic Mice. Int Immunopharmacol (2018) 63:227–38. doi: 10.1016/j.intimp.2018.07.027 30107367

[59] Masselli E Pozzi G Vaccarezza M Mirandola P Galli D Vitale M. ROS in Platelet Biology: Functional Aspects and Methodological Insights. Int J Mol Sci (2020) 21(14):692–8. doi: 10.3390/ijms21144866 PMC7402354 32660144

[60] Habashy WS Milfort MC Rekaya R Aggrey SE. Expression of Genes That Encode Cellular Oxidant/Antioxidant Systems Are Affected by Heat Stress. Mol Biol Rep (2018) 45(3):389–94. doi: 10.1007/s11033-018-4173-0 29619655

[61] Geng Y Li R He SX Yang HH Deng QT Shao XY. Dexmedetomidine Attenuates Acute Lung Injury Induced by Heatstroke and Improve Outcome. Shock (2018) 10(5):782–803. doi: 10.1097/shk.0000000000001289 30475328

[62] Gross ER Zambelli VO Small BA Ferreira JC Chen CH Mochly-Rosen D. A Personalized Medicine Approach for Asian Americans With the Aldehyde Dehydrogenase 2*2 Variant. Annu Rev Pharmacol Toxicol (2015) 55:107–27. doi: 10.1146/annurev-pharmtox-010814-124915 PMC4435945 25292432

[63] Zhong H Yin H. Role of Lipid Peroxidation Derived 4-Hydroxynonenal (4-HNE) in Cancer: Focusing on Mitochondria. Redox Biol (2015) 4:193–9. doi: 10.1016/j.redox.2014.12.011 PMC4803793 25598486

[64] Chen C-H Sun L Mochly-Rosen D. Mitochondrial Aldehyde Dehydrogenase and Cardiac Diseases. Cardiovasc Res (2010) 88(1):51–7. doi: 10.1093/cvr/cvq192 PMC2936126 20558439

[65] Zhong S Li L Zhang Y-L Zhang L Lu J Guo S. Acetaldehyde Dehydrogenase 2 Interactions With LDLR and AMPK Regulate Foam Cell Formation. J Clin Invest (2018) 129(1):389–94. doi: 10.1172/JCI122064 PMC6307968 30375985

[66] Guo YJ Chen L Bai YP Li L Sun J Zhang GG. The ALDH2 Glu504Lys Polymorphism Is Associated With Coronary Artery Disease in Han Chinese: Relation With Endothelial ADMA Levels. Atherosclerosis (2010) 211(2):545–50. doi: 10.1016/j.atherosclerosis.2010.03.030 20417517

[67] Gong D Zhang L Zhang Y Wang F Zhou X Sun H. East Asian Variant of Aldehyde Dehydrogenase 2 Is Related to Worse Cardioprotective Results After Coronary Artery Bypass Grafting. Interact Cardiovasc Thorac Surg (2019) 28(1):79–84. doi: 10.1093/icvts/ivy204 29982537

[68] Kim J Chen CH Yang J Mochly-Rosen D. Aldehyde Dehydrogenase 2*2 Knock-in Mice Show Increased Reactive Oxygen Species Production in Response to Cisplatin Treatment. J BioMed Sci (2017) 24(1):33. doi: 10.1186/s12929-017-0338-8 28532411 PMC5439151

[69] Kwon HJ Won YS Park O Chang B Duryee MJ Thiele GE. Aldehyde Dehydrogenase 2 Deficiency Ameliorates Alcoholic Fatty Liver But Worsens Liver Inflammation and Fibrosis in Mice. Hepatology (2014) 60(1):146–57. doi: 10.1002/hep.27036 PMC4077916 24492981

[70] Pan C Xing JH Zhang C Zhang YM Zhang LT Wei SJ. Aldehyde Dehydrogenase 2 Inhibits Inflammatory Response and Regulates Atherosclerotic Plaque. Oncotarget (2016) 7(24):35562–76. doi: 10.18632/oncotarget.9384 PMC5094945 27191745

[71] Zhang P Xu D Wang S Fu H Wang K Zou Y. Inhibition of Aldehyde Dehydrogenase 2 Activity Enhances Antimycin-Induced Rat Cardiomyocytes Apoptosis Through Activation of MAPK Signaling Pathway. BioMed Pharmacother (2011) 65(8):590–3. doi: 10.1016/j.biopha.2009.12.001 21123025

[72] Chen HW Kuo HT Hwang LC Kuo MF Yang RC. Proteomic Alteration of Mitochondrial Aldehyde Dehydrogenase 2 in Sepsis Regulated by Heat Shock Response. Shock (2007) 28(6):710–6. doi: 10.1097/shk.0b013e318050c8c2 17607160

[73] Hu XY Fang Q Ma D Jiang L Yang Y Sun J. Aldehyde Dehydrogenase 2 Protects Human Umbilical Vein Endothelial Cells Against Oxidative Damage and Increases Endothelial Nitric Oxide Production to Reverse Nitroglycerin Tolerance. Genet Mol Res (2016) 15(2):gmr7822. doi: 10.4238/gmr.15027822 27323160

[74] Li SY Gomelsky M Duan J Zhang Z Gomelsky L Zhang X. Overexpression of Aldehyde Dehydrogenase-2 (ALDH2) Transgene Prevents Acetaldehyde-Induced Cell Injury in Human Umbilical Vein Endothelial Cells: Role of ERK and P38 Mitogen-Activated Protein Kinase. J Biol Chem (2004) 279(12):11244–52. doi: 10.1074/jbc.M308011200 14722101

[75] Zhong Z Ye S Xiong Y Wu L Zhang M Fan X. Decreased Expression of Mitochondrial Aldehyde Dehydrogenase-2 Induces Liver Injury *via* Activation of the Mitogen-Activated Protein Kinase Pathway. Transpl Int (2016) 29(1):98–107. doi: 10.1111/tri.12675 26404764

[76] Xiao Q Weiner H Crabb DW. The Mutation in the Mitochondrial Aldehyde Dehydrogenase (ALDH2) Gene Responsible for Alcohol-Induced Flushing Increases Turnover of the Enzyme Tetramers in a Dominant Fashion. J Clin Invest (1996) 98(9):2027–32. doi: 10.1172/JCI119007 PMC507646 8903321

[77] Jin S Chen J Chen L Histen G Lin Z Gross S. ALDH2(E487K) Mutation Increases Protein Turnover and Promotes Murine Hepatocarcinogenesis. Proc Natl Acad Sci USA (2015) 112(29):9088–93. doi: 10.1073/pnas.1510757112 PMC4517197 26150517

[78] Kang P Wang J Fang D Fang T Yu Y Zhang W. Activation of ALDH2 Attenuates High Glucose Induced Rat Cardiomyocyte Fibrosis and Necroptosis. Free Radic Biol Med (2020) 146:198–210. doi: 10.1016/j.freeradbiomed.2019.10.416 31689484

[79] Xu Y Yuan Q Cao S Cui S Xue L Song X. Aldehyde Dehydrogenase 2 Inhibited Oxidized LDL-Induced NLRP3 Inflammasome Priming and Activation *via* Attenuating Oxidative Stress. Biochem Biophys Res Commun (2020) 529(4):998–1004. doi: 10.1016/j.bbrc.2020.06.075 32819611

[80] Liu M Lu S He W Zhang L Ma Y Lv P. ULK1-Regulated Autophagy: A Mechanism in Cellular Protection for ALDH2 Against Hyperglycemia. Toxicol Lett (2018) 283:106–15. doi: 10.1016/j.toxlet.2017.11.008 29128638

[81] Comellas AP Briva A Dada LA Butti ML Trejo HE Yshii C. Endothelin-1 Impairs Alveolar Epithelial Function *via* Endothelial ETB Receptor. Am J Respir Crit Care Med (2009) 179(2):113–22. doi: 10.1164/rccm.200804-540OC PMC2633058 18948426

[82] Ji E Jiao T Shen Y Xu Y Sun Y Cai Z. Molecular Mechanism of HSF1-Upregulated ALDH2 by PKC in Ameliorating Pressure Overload-Induced Heart Failure in Mice. BioMed Res Int (2020) 2020:3481623. doi: 10.1155/2020/3481623 32626739 PMC7313111

[83] Yang LL Chen H Wang J Xia T Sun H Yuan CH. 4-HNE Induces Apoptosis of Human Retinal Pigment Epithelial Cells by Modifying Hsp70. Curr Med Sci (2019) 39(3):442–8. doi: 10.1007/s11596-019-2057-8 31209817

[84] Galligan JJ Fritz KS Backos DS Shearn CT Smathers RL Jiang H. Oxidative Stress-Mediated Aldehyde Adduction of GRP78 in a Mouse Model of Alcoholic Liver Disease: Functional Independence of ATPase Activity and Chaperone Function. Free Radic Biol Med (2014) 73:411–20. doi: 10.1016/j.freeradbiomed.2014.06.002 PMC4395467 24924946

