# Supplementary material for: Pharmacological Activation Of Aldehyde Dehydrogenase 2 Protects Against Heatstroke-Induced Acute Lung Injury by Modulating Oxidative Stress and Endothelial Dysfunction
Source: Front Immunol. 2021 Oct 26;12:740562. doi: 10.3389/fimmu.2021.740562 (PMC8576434; doi:10.3389/fimmu.2021.740562)
Supplement: Supplementary Figure 1 — The core temperature of the experimental animals. Body temperature changes in WT and ALDH2*2 KI mice subjected to WBH. [file DataSheet_1.pdf]

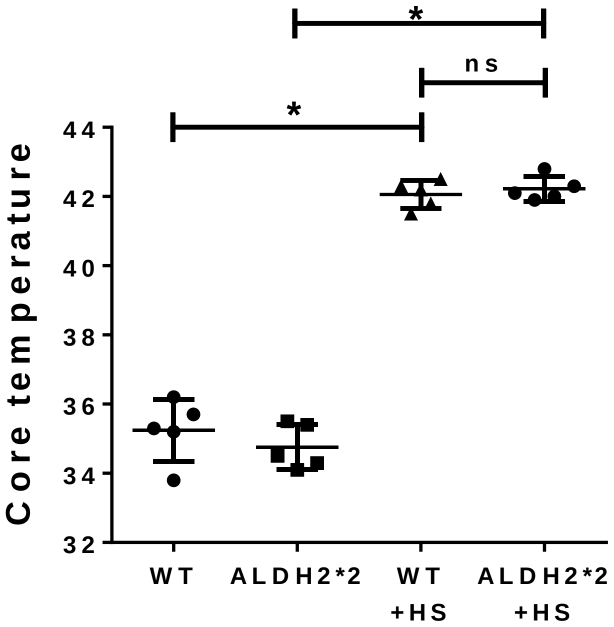

Figure S1. Body temperature changes of the WT and ALDH2*2 KI mice subjected to WBH.

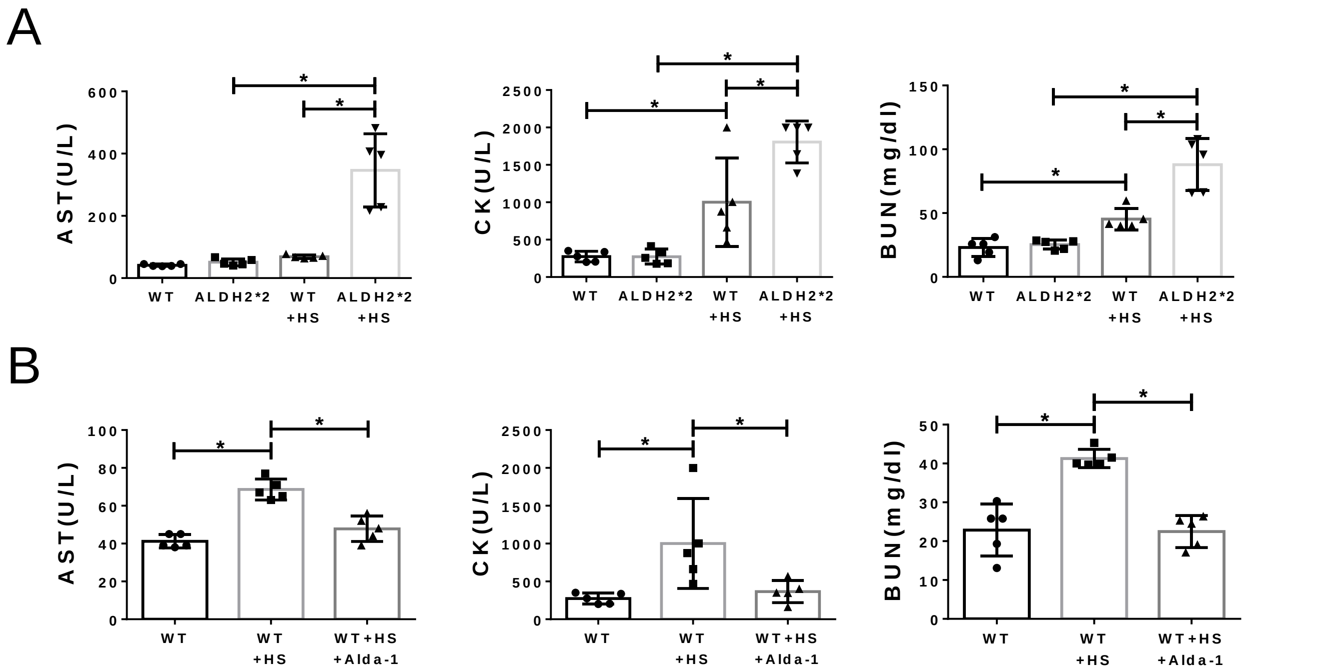

Figure S2. The serum levels of AST, CK, BUN in experimental animals. The serum levels of AST, CK, and BUN in experimental animals. ALDH2*2 KI mice and their littermates **(A)** and Alda-1-treated mice **(B)** subjected to WBH.

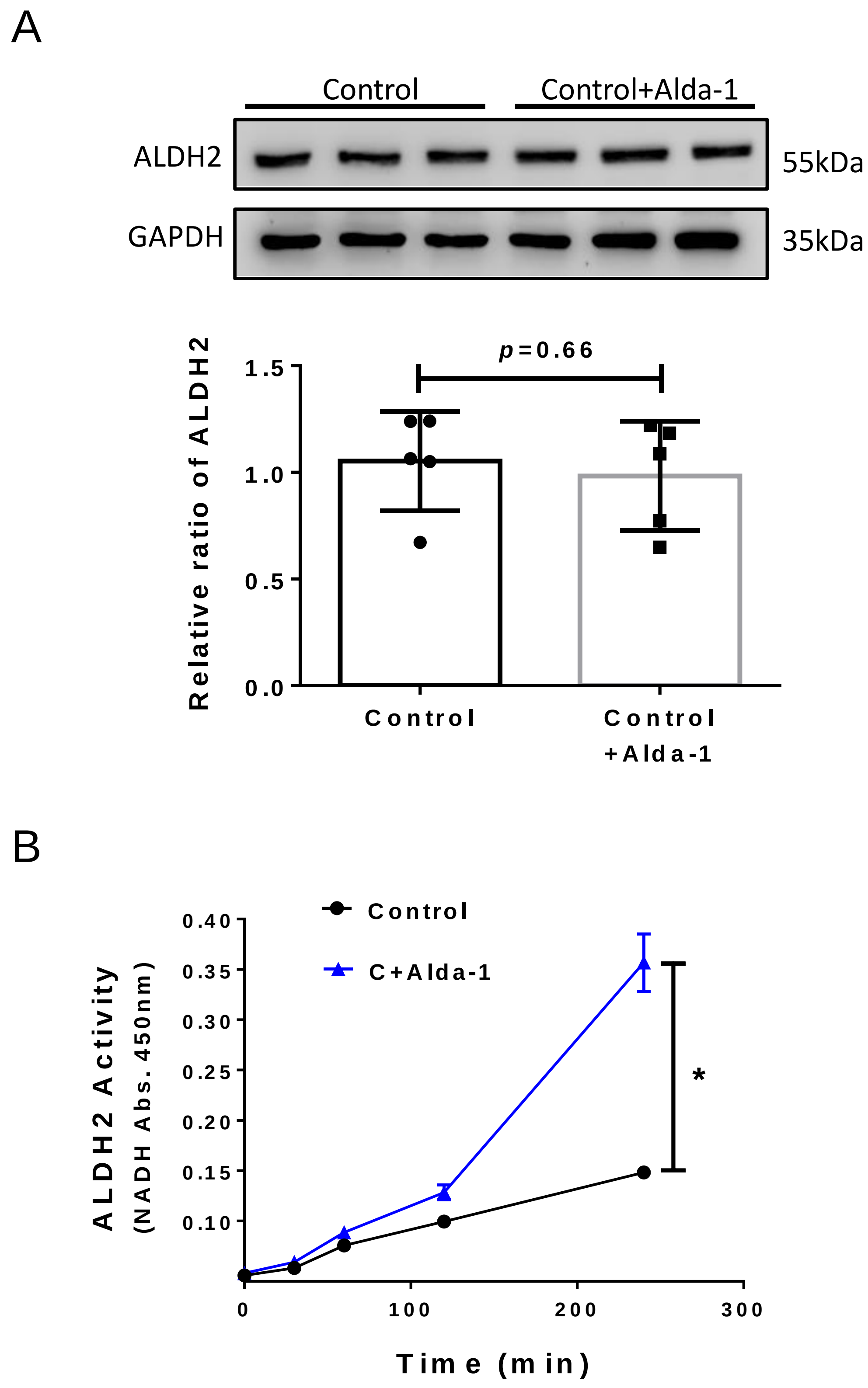

Figure S3. Alda-1 increased ALDH2 activities but not expression in HUVECs. Alda-1 increased the ALDH2 activity but not its expression in HUVECs. **(A)** ALDH2 protein expression was measured by immunoblotting (*n* = 5). **(B)** ALDH2 activity was measured by NADH production as determined by the O.D. absorbance at 450 nm in a microplate reader (*n* = 5).
